# Propensity-Matched Case Control Study of Anti-Interleukin-23 Therapies in Clostridioides difficile Infection

**DOI:** 10.21203/rs.3.rs-10060050/v1

**Published:** 2026-07-03

**Authors:** Gregory R. Madden, Jennie Z. Ma

**Affiliations:** University of Virginia; University of Virginia

**Keywords:** Clostridioides difficile, C. difficile infection, monoclonal antibody, interleukin-23, IL-23, Th17 immunity, retrospective study

## Abstract

**Background:**

*Clostridioides difficile* infection remains a major cause of infectious morbidity and mortality, largely driven by a dysregulated, toxin-mediated host immune response that current pathogen-targeted therapies fail to address. Interleukin-23-driven Th17 responses and subsequent neutrophil activation play a major role in disease severity and intestinal injury. Observational data suggest that targeting this pathway with anti-IL-23 monoclonal antibodies (therapies already used for inflammatory bowel disease, psoriasis) may significantly reduce mortality in these patients.

**Methods:**

We conducted a retrospective cohort study of adult patients hospitalized with *C. difficile* infection using the Epic Cosmos electronic health record database (> 300 million patients across 1,949 healthcare organizations). Propensity score matching (10:1) was used to balance baseline demographic and clinical covariates between patients exposed an available anti-IL-23 monoclonal antibody therapy (ustekinumab, guselkumab, tildrakizumab, risankizumab, mirikizumab) within 90 days prior to diagnosis and unexposed controls. The primary outcome was 30-day all-cause mortality, evaluated using a multivariable Cox proportional hazards regression model, adjusted by ATLAS severity.

**Results:**

The propensity-matched cohort included 328 index cases of *C. difficile* infection exposed to anti-IL-23 within 90-days and 3,155 controls. Anti-IL-23 exposure was associated with a significantly reduced hazard of 30-day mortality (HR 0.341, 95% CI 0.121–0.962, *P* = 0.042). Heterogeneity of treatment effect analysis revealed a statistically significant positive interaction between baseline ATLAS severity and anti-IL-23 efficacy (*P* = 0.029). No significant difference was observed in 180-day recurrent infection-free survival between groups (HR 0.808, 95% CI 0.514–1.27, *P* = 0.355).

**Conclusions:**

Antecedent anti-IL-23 therapy is associated with a significant reduction in 30-day mortality following *C. difficile* infection. These findings support the concept that inhibiting IL-23 mediated inflammation alongside standard anti-*C. difficile* antibiotics may improve outcomes in *C. difficile* infection. The significant interaction with baseline severity suggests that the clinical utility of this immunomodulatory therapy may lie in high-risk patient phenotypes. Further prospective studies are warranted to confirm these observations and evaluate the clinical role for IL-23 inhibition in *C. difficile* infection.

## Introduction

*C. difficile* produces a diarrheal toxin which stimulates a strong pathogenic immune response, resulting in substantial mortality (> 18% in severe cases) [1], morbidity, increased hospital length of stay, and considerable healthcare costs [2]. A fifth or more of *C. difficile* infection survivors will suffer subsequent recurrent infection, which carries an additional (1.3-fold) risk of death [3].

Increasing evidence suggests that a patient’s immune response to *C. difficile* infection determines their clinical outcome [4]. However, existing options for severe or treatment-refractory *C. difficile* infection target the pathogen itself or restore colonization resistance but do little to modulate the dysregulated host immune response; these include changing or combining anti-*C. difficile* antibiotics (which risks further microbiome disruption), their administration route (i.e., per rectum/ileostomy), intestinal surgery (high morbidity) [5], and/or early fecal microbiota transplant (rarely done during acute infection due to limited evidence, safety concerns [6], and logistical/regulatory barriers) [7]. Targeted immunotherapy could help meet the need for more effective *C. difficile* treatment as an adjunct to anti-*C. difficile* antibiotics to mitigate the underlying toxin-mediated immune response while sparing protective gut microbes.

IL-23-mediated Th17 immunity plays an important role in *C. difficile*-mediated neutrophil activation, leading to severe outcomes [8] During *C. difficile* colitis, IL-23-positive immune cells infiltrate the colon [9] and higher serum IL-23 positively correlates with disease severity [10]. We previously reported that anti-IL-23 monoclonal antibody therapies (ustekinumab, guselkumab, tildrakizumab, risankizumab, or mirikizumab) prior to a hospitalized acute *C. difficile* infection was associated with an 82.5% reduction in 30-day mortality in an aggregated propensity-matched multicenter retrospective case control study across 90 healthcare organizations (TriNetX) [11]. To validate and extend these results, we conducted a larger retrospective study using a separate database with access to more granular, line-level data involving institutions utilizing the Epic Cosmos electronic health record system.

## Methods

Data used in this study came from Epic Cosmos, a dataset created in collaboration with a community of Epic health systems representing more than 300 million patient records from over 1,949 hospitals and 47,000 clinics from all 50 U.S. states, D.C., Lebanon, and Saudi Arabia at the time of query in May 2026. Hospitalized *C. difficile* infection cases were identified that occurred between January 1, 2018 to March 1, 2026. Hospitalized *C. difficile* infection cases were defined as inpatient encounters with a positive *C. difficile* test (polymerase chain reaction or enzyme immunoassay, with the earliest positive stool test marked as the day of diagnosis) coupled with receipt of first-line guideline-recommended anti-*C. difficile* antibiotics [5] (oral vancomycin or fidaxomicin) within 48 hours of diagnosis. Patients treated with metronidazole monotherapy were not included due to concerns over reduced efficacy in *C. difficile* infection and to avoid misdiagnoses due to use of metronidazole for non-*C. difficile* indications [1]. For individual patient identifiers associated with multiple episodes meeting the *C. difficile* infection definition, the first (‘index’) case occurring within the database was used and subsequent episodes were marked as “recurrent” infections. Patients were excluded from the analysis if Epic Cosmos’ registry used for longitudinal patient tracking (ActivePatientRegistryDataMartX, or APRDMX) failed to indicate that the index diagnosis occurred when the patient had at least 2 face-to-face encounters within a 2-year window, indicating ongoing engagement with the healthcare system and sufficient longitudinal data capture. Anti-IL-23 therapy was defined as receipt of at least 1 dose of commercially-available anti-IL-23 monoclonal antibody agents (ustekinumab, guselkumab, tildrakizumab, risankizumab, or mirikizumab) within 90 days prior to the index *C. difficile* infection diagnosis, from recorded medication administrations or dispensations. Psoriasis and/or inflammatory bowel disease (current accepted indications for use of anti-IL-23 medications) were identified using diagnosis events active on the diagnosis date. Hospital length of stay was defined as the total duration, in days, from admission to discharge during the index *C. difficile* infection encounter.

A logistic regression model was used to calculate a propensity score (i.e., probability) of receiving anti-IL-23 for each patient, based on baseline demographic and clinical covariates known to influence treatment assignment (psoriasis, inflammatory bowel disease) and/or adverse *C. difficile* infection outcomes, including age, sex [12], baseline laboratory values (white blood cell count (WBC), albumin, creatinine), intensive care unit location (on day of diagnosis), vasopressor support, immunosuppression, proton pump inhibitor use (within 90 days) [13], and non-*C. difficile* antibiotic exposure (as defined by Miller et al. [14]). Immunosuppression was defined as receipt of corticosteroids (60mg prednisone or equivalent), tacrolimus, cyclosporine, mycophenolate, azathioprine, or methotrexate within 90 days prior to diagnosis. Vasopressors were defined as receipt of intravenous norepinephrine, epinephrine, vasopressin, phenylephrine, dobutamine, or dobutamine on the day of diagnosis. Baseline laboratory data were defined as the most recent measurement available on or prior to the diagnosis date. Cases with unavailable baseline lab tests (21,379/22 WBC, 7,307/11 creatinine, and 37,704/46 albumin measurements for control/anti-IL-23 groups, respectively) were imputed as median values from the entire cohort. Propensity score matching was performed using a nearest-neighbor algorithm without replacement. Each patient exposed to anti-IL-23 was matched to up to ten unexposed control patients (a 10:1 ratio) based on the calculated propensity scores to optimize baseline covariate balance.

The primary endpoint was 30-day all-cause mortality following index *C. difficile* infection diagnosis, analyzed as a time-to-event outcome. Survival probabilities were estimated using the Kaplan–Meier method and the effect of anti-IL-23 exposure was estimated using the Cox proportional hazards model. Death events were ascertained using the available death date field and follow-up time was censored at 30 days after diagnosis or at the administrative end of data availability, whichever occurred first. To achieve a doubly robust estimate of the treatment effect, baseline ATLAS score (a validated measure of baseline *C. difficile* mortality risk by Miller et al. [14, 15]) was included as an adjustment covariate in the final Cox proportional hazards model. To account for clustering with matched sets in the propensity-matched cohort, we conducted sensitivity analyses using Cox proportional hazards models stratified by matched set, both without and with adjustment for ATLAS score.

To evaluate whether treatment efficacy varied based on baseline disease severity, a heterogeneity of treatment effect analysis was performed as a secondary evaluation by introducing a multiplicative interaction term between anti-IL-23 exposure and the baseline ATLAS score (0–10) in the primary Cox model. Other secondary outcomes included recurrent *C. difficile* infection-free survival and hospital length of stay. The recurrent *C. difficile* infection-free survival is a composite time-to-event outcome, with the event occurring at the earliest of either subsequent positive *C. difficile* laboratory test coupled with anti-*C. difficile* treatment or death within 180 days of the index *C. difficile* infection. The composite outcome was evaluated using a univariable Cox proportional hazards regression model. Cosmos follows the Centers for Medicare and Medicaid Services Cell Size Suppression Policy for all published results and therefore, counts of 10 or fewer are masked as < 11 and source data are not available to be shared for privacy reasons. Analyses were performed using statistical software R, version 4.5.1 (R Core Team, Vienna, Austria) and the following key R packages: comorbidity [16], MatchIt [17], survival [18], and emmeans.

## Results

A total of 190,372 index hospitalized *C. difficile* infection cases were identified without anti-IL-23 treatment and 330 cases that received an anti-IL-23 medication within the preceding 90 days (179 ustekinumab, 34 guselkumab, < 11 tildrakizumab, 113 risankizumab, < 11 mirikizumab). Of the original 330 treated patients, 2 were excluded from the propensity-matched cohort due to a lack of comparable controls within the prespecified caliper distance (0.2 standard deviations of the logit of the propensity score), totaling 328. Similarly, the matched control group (n = 3,155) fell short of the 10:1 ratio because the pool of acceptable matches was exhausted. Baseline characteristics, laboratory measurements, and comorbid conditions for the full and propensity-matched cohorts are shown in Table 1.

**Figure 1:**
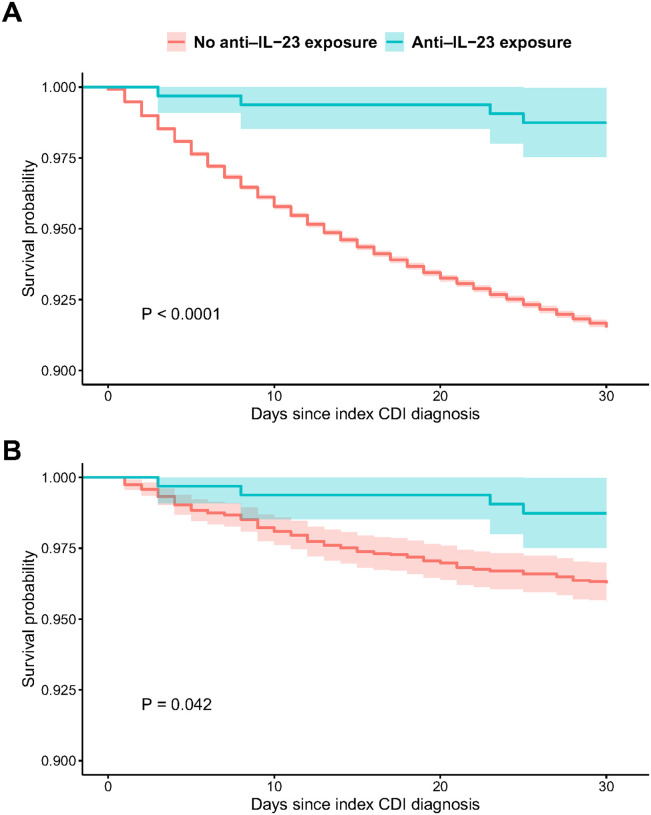
Kaplan Meier Survival Curves A) Unmatched Cohort, B) Propensity-Matched Cohort. Shaded areas represent the 95% confidence interval. Abbreviations: CDI, C. difficile infection.

The primary time-to-event analysis using a Cox proportional hazards model, adjusted for baseline severity, showed that anti-IL-23 exposure was associated with a significantly reduced hazard of 30-day mortality in the propensity-matched cohort (Hazard Ratio (HR) 0.341, 95% Confidence Interval (CI) 0.121–0.962, *P* = 0.042). ATLAS severity remained a significant predictor of mortality (HR 1.850, 95% CI 1.706–2.005, *P* < 0.001). Kaplan Meier survival curves of the unmatched and matched cohorts are shown in Fig. 1. As a sensitivity analysis, stratified Cox regression by matched sets yielded similar treatment effect estimates in magnitude to those from the primary analysis. Specifically, the hazard ratio of anti-IL-23 was 0.35 (95% CI 0.131–0.934, *P* = 0.036) without adjustment for ATLAS score and 0.40 (95% CI 0.154–1.057, *P* = 0.065) after adjustment. Overall, the protective treatment effect of anti-IL-23 remained consistent, indicating that the findings are robust despite the reduced effective sample size with matched-set stratification.

**Figure 2:**
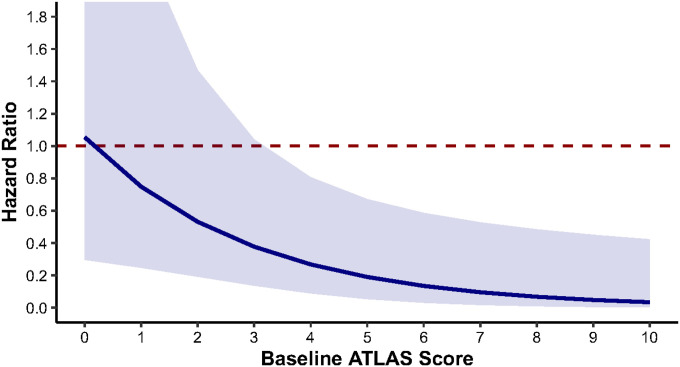
Heterogeneity of Treatment Effect Analysis of Anti-IL-23 on 30-day Mortality Across Baseline ATLAS Severity Scores. The solid blue line represents the estimated marginal hazard ratio comparing anti-IL-23 pre-treatment to propensity-matched unexposed controls across baseline ATLAS scores 0–10. The shaded region denotes the 95% confidence interval. The horizontal dashed red line indicates a hazard ratio of 1.0 (the line of no effect).

**Figure 3:**
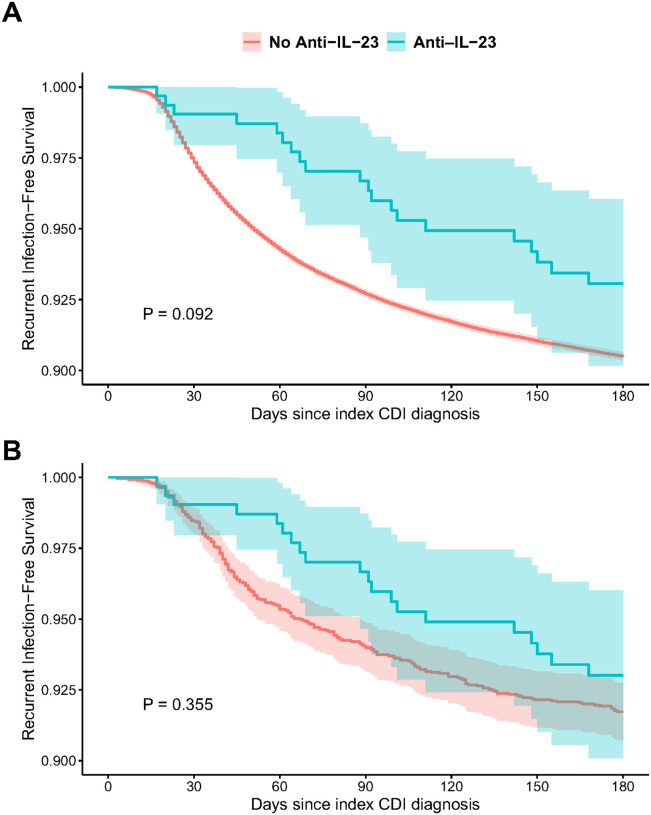
Recurrence-Free Survival Curves Shaded areas represent the 95% confidence interval. Abbreviations: CDI, C. difficile infection.

The secondary analysis evaluating treatment effect heterogeneity across ATLAS score levels revealed a significant interaction between anti-IL-23 exposure and baseline ATLAS score (interaction HR 0.710, *P* = 0.0292). To interpret this interaction, we estimated the specific hazard ratios of anti-IL-23 therapy at each point along the 0–10 ATLAS severity scale (Table 2); the resulting marginal effects are illustrated in Fig. 2. 180-day recurrent infection-free survival was observed in 22/328 (6.7%) of patients that received anti-IL-23 compared to 240/3,155 (7.6%) of unexposed matched controls. In a corresponding univariable Cox proportional hazards regression model (Fig. 3), anti-IL-23 exposure was not associated with statistically significant improvement in long-term recurrence-free survival (HR 0.808, 95% CI 0.514–1.27, *P* = 0.355). Mean hospital length of stay was 6.83 days (standard deviation (SD) 8.57) among anti-IL-23 patients compared to 8.01 days (SD 8.39) for controls (*P* = 0.016).

## Conclusions

In this large, propensity-matched retrospective cohort study, we found that antecedent exposure to anti-IL-23 monoclonal antibodies was associated with a significant reduction in 30-day all-cause mortality among hospitalized patients with *C. difficile* infection. We identified a severity-dependent interaction demonstrating that the survival benefit of IL-23 inhibition becomes markedly more pronounced as the patient’s baseline severity increases, particularly with an ATLAS score > 3. Despite the early mortality benefit, anti-IL-23 therapy was associated with significantly reduced hospital length of stay (by approximately 1.2 days) but did not significantly alter the risk of long-term *C. difficile* recurrence.

These findings validate and extend our previous reports of a survival signal associated with IL-23 blockage in *C. difficile* infection [11, 19]. The biological rationale for anti-IL-23 in *C. difficile* infection is that bacterial TcdB toxin triggers a host immune response which drives IL-23 production and subsequent neutrophil infiltration into the colonic mucosa, exacerbating tissue damage and systemic toxicity [8, 9]. By interrupting this upstream inflammatory cascade, anti-IL-23 agents appear to mitigate the collateral damage by the host immune system.

While earlier retrospective clinical investigations relied on aggregative, administrative diagnostic codes and basic demographic matching, the current study leverages highly-granular, line-level clinical data. Epic Cosmos has major advantages over other large administrative databases which often lack of direct access to patient-level information and/or lack specific laboratory tests and longitudinal data. Billing/coding data are often used define *C. difficile* infection is far from ideal [20]. We identified hospitalized *C. difficile* infection cases using specific stool lab test results coupled with physician ordered, guideline-recommended anti-*C. difficile* antibiotic treatment as a clinically-relevant cohort. By rigorously adjusting for diagnoses associated with anti-IL-23 use, intensive care unit admission, immunosuppression, vasopressors, and individual components of the validated ATLAS severity criteria, this study provides stronger evidence for the role of the IL-23/Th17 axis in determining acute clinical outcomes.

This study has important limitations. A very large dataset such as Epic Cosmos is susceptible to the Big Data Paradox whereby a very large sample size leads to very precise but incorrect conclusions, possibly due to patient underrepresentation (e.g., patients who do not seek healthcare or underrepresented demographics) biased inference. Recurrent infections were identifiable only if subsequent *C. difficile* infection encounters appeared within the Epic Cosmos database and thus were likely undercounted. Although a data completeness proxy was used (APRDMX), multicenter databases are prone to missing or heterogeneous data across organizations.

## Figures and Tables

**Table 1:** Baseline Characteristics and Outcomes of Unmatched and Propensity-Matched Cohorts

Characteristic	Unmatched Cohort			Matched Cohort		
	Control (n = 190,372)	Treated (n = 330)	*P*	Control (n = 3,155)	Treated (n = 328)	*P*
Age (mean years (SD))	66.97 (18.0)	47.15 (21.0)	< 0.001	47.61 (22.3)	47.22 (21.0)	0.763
Male sex	82,222 (43.2%)	152 (46.1%)	0.771	1,476 (46.8%)	151 (46.0%)	0.842
Ethnicity			0.354			0.033
Hispanic/Latino	11,864 (6.2%)	18 (5.5%)		243 (7.7%)	17 (5.2)	
Not Hispanic/Latino	158,283 (83.1%)	284 (86.1%)		2,535 (80.3%)	283 (86.3%)	
Unspecified	20,225 (10.6%)	28 (8.5%)		377 (11.9%)	28 (8.5%)	
Psoriasis	1,728 (0.9%)	54 (16.4%)	< 0.001	141 (4.5%)	52 (15.9%)	< 0.001
Inflammatory Bowel Disease	14,519 (7.6)	264 (80%)	< 0.001	2,733 (86.6%)	262 (79.9%)	0.001
WBC (median cells × 10^3^/μL (IQR)	10.54 (7.3–15.2)	10.1 (7.26–13.2)	0.034	10.54 (7.4–13.8)	10.10 (7.2–13.2)	0.288
Creatinine (median (IQR))	1.03 (0.73–1.73)	0.80 (0.62–1.06)	< 0.001	0.81 (0.64–1.1)	0.80 (0.63–1.06)	0.812
Albumin (median g/dL (IQR))	3.00 (2.70–3.40)	3.2 (2.90–3.80)	< 0.001	3.00 (2.8–3.6)	3.2 (2.9–3.8)	0.001
Intensive Care	29,771 (15.6%)	26 (7.9%)	< 0.001	135 (4.3%)	13 (4.0%)	1.000
Vasopressors (%)	13,126 (6.9%)	13 (3.9%)	0.045	135 (4.3%)	13 (4.0)	> 0.99
Proton Pump Inhibitor use (within 90d, %)	46,668 (24.5%)	71 (21.5)	0.230	650 (20.6%)	70 (21.3%)	0.808
Immunosuppression within 90 days	20,354 (10.7%)	88 (26.7%)	< 0.001	780 (24.7%)	86 (26.2)	0.596
ATLAS score (0–10, mean (SD))	2.98 (1.68)	1.65 (1.50)	< 0.001	1.88 (1.53)	1.66 (1.50)	0.015
Anti-*C. difficile* Treatment			0.003			0.024
oral vancomycin	166,081 (87.1%)	269 (81.5%)		2,718 (86.1%)	267 (81.4%)	
fidaxomicin	24,291 (12.9)	61 (18.5%)		437 (13.9%)	61 (18.6%)	

Differences in baseline characteristics between the groups were evaluated using Pearson’s chi-squared test or Fisher’s exact test for categorical variables, and the independent-samples Student’s t-test or Wilcoxon rank-sum test for continuous variables, as appropriate for the data distribution (no adjustment for multiple comparisons).

Abbreviations: SD, standard deviation; IQR, interquartile range; WBC, White Blood Cell Count; d, days, ATLAS, (Age, Treatment, Leukocyte count, Albumin, Serum creatinine) C. difficile infection severity score.

**Table 2:** Conditional Treatment Effects of Anti-IL-23 Therapy by Baseline Disease Severity

Baseline ATLAS Score	Hazard Ratio (95% CI)	*P*
0	1.05 (0.30–3.75)	0.935
1	0.75 (0.25–2.26)	0.608
2	0.53 (0.19–1.47)	0.223
3	0.38 (0.13–1.06)	0.060
4	0.27 (0.09–0.81)	0.019
5	0.19 (0.05–0.67)	0.010
6	0.14 (0.03–0.59)	0.008
7	0.10 (0.02–0.53)	0.007
8	0.07 (0.01–0.48)	0.007
9	0.05 (0.00–0.45)	0.008
10	0.03 (0.00–1.42)	0.008

Hazard ratios (HR) and 95% confidence intervals (CI) represent the relative risk of 30-day all-cause mortality for patients with antecedent anti-IL-23 exposure compared to unexposed matched controls at each baseline ATLAS score. Estimates were derived using marginal contrasts from the primary Cox proportional hazards model, which included a treatment-by-severity interaction term. A HR < 1.00 indicates a protective survival benefit associated with anti-IL-23 therapy.

Abbreviations: ATLAS, (Age, Treatment, Leukocyte count, Albumin, Serum creatinine), CI, confidence interval; HR, hazard ratio.

## Data Availability

The data that support the findings of this study are available from Epic Cosmos, but restrictions apply to the availability of these data, which were used under license for the current study and are not publicly available. Data and code are, however, available from the authors upon reasonable request and with permission of Epic Cosmos.
